# Independently tunable electromagnetically induced transparency effect and dispersion in a multi-band terahertz metamaterial

**DOI:** 10.1038/s41598-019-54414-5

**Published:** 2019-12-02

**Authors:** Rakesh Sarkar, Dipa Ghindani, Koijam Monika Devi, S. S. Prabhu, Amir Ahmad, Gagan Kumar

**Affiliations:** 1 Department of Physics, Indian Institute of Technology Guwahati, Guwahati, 781039 Assam India; 2 Tata Institute of Fundamental Research, Navy Nagar, Colaba, Mumbai, 400005 India; 3 College of Information Technology, United Arab Emirates University, Al Ain, 15551 United Arab Emirates

**Keywords:** Metamaterials, Slow light, Terahertz optics

## Abstract

In this article, we experimentally and numerically investigate a planar terahertz metamaterial (MM) geometry capable of exhibiting independently tunable multi-band electromagnetically induced transparency effect (EIT). The MM structure exhibits multi-band EIT effect due to the strong near field coupling between the bright mode of the cut-wire (CW) and dark modes of pair of asymmetric double C resonators (DCRs). The configuration allows us to independently tune the transparency windows which is challenging task in multiband EIT effect. The independent modulation is achieved by displacing one DCR with respect to the CW, while keeping the other asymmetric DCR fixed. We further examine steep dispersive behavior of the transmission spectra within the transparency windows and analyze slow light properties. A coupled harmonic oscillator based theoretical model is employed to elucidate as well as understand the experimental and numerical observations. The study can be highly significant in the development of multi-band slow light devices, buffers and modulators.

## Introduction

The phenomenon of electromagnetically induced transparency (EIT) has garnered a lot of attraction in the scientific community over the last few decades. EIT, which is a quantum phenomenon, leads to elimination of absorption for a narrow region within a wider absorption spectrum^1–4^. EIT occurs as a result of destructive interference between two transition pathways of an atomic medium and gives rise to a significant change in the dispersive properties of the medium within the narrow transparency region. EIT phenomenon has been studied using different atomic medium for the generation of self-enhanced nonlinearity^5^, ultraslow group velocity and storage of light^6^. In the last decade, the scientific community has managed to demonstrate an analogue of EIT in artificially engineered materials known as the metamaterials (MMs)^7–19^. One advantage of studying EIT in metamaterials is the fact that the effect can occur even at room temperature. In metamaterials (MMs), EIT usually occurs either between near field coupled bright-bright modes^13,20^ or between bright-dark modes^4,21–23^. The bright mode is the mode that strongly couples with the incident electric field and exhibits broader resonance having low quality factor. On the other hand, the dark mode either does not couple or couples very weakly with the incident electric field and results in sharper resonance exhibiting higher quality factor^24–27^. When these modes have approximately same resonance frequencies, the destructive interference leads to a narrow transparency window which has potential applications in the development of sensing and slow light devices. Various MM structures such as metal strips, coupled SRRs etc. have been reported to exhibit EIT effect from optical to infrared frequencies^28–33^.

In recent years, multi-band transparency effect has been able to grab considerable attention. In this direction, multi-band EIT has been greatly investigated using different MM structures. Multi-band EIT effect has been reported to occur due to the coupling between bright and dark modes or bright, dark and quasi dark modes. Quasi dark modes can couple with the incident electric field, however as compared to the bright mode it has weaker confinement. Several MM configurations have been exploited to exhibit multi-band EIT effect in the microwave^34,35^, infrared^36^ as well as in the terahertz frequency regimes^37–39^. Zhang *et al*. investigated a MM geometry comprising of three meta-atoms acting as bright, dark and quasi dark modes that exhibits dual-band EIT effect in the terahertz frequency regime^37^. Kim *et al*. numerically investigated a MM structure comprising of multi rings that exhibits multi peak EIT effect in the terahertz frequency regime^38^. He *et al*. reported a planar MM composed of a gold bar and two gold wire pairs that exhibits dual-spectral EIT effect due to the coupling between bright and dark modes^40^. Multi-band transparency effect effect has also been realized in other MM structures such as multilayered coupled meta-atoms, four-fold symmertric structures and metal graphne based structures^41–43^. The multi-band EIT effect has potential in sensing, slow light systems and storage applications over the multiple frequency bands.

Recently, efforts have also been made to modulate the transparency windows in multiband transparency spectrum. Devi *et al*. investigated dual-band EIT effect and it’s modulation effect in a terahertz MM structure comprising of an inner circular and outer asymmetric two-gap circular split ring resonators^44^. Tang *et al*. investigated the dual-band EIT effect and also studied modulation effect of transparency windows by changing the structural parameters in a MM structure compring of closed ring and a square patch^45^. Also graphene based MM structures have been reported earlier in which dynamic modulation of multispectral plasmon induced transparency effect has been achieved^46–48^. In these investigations, the focus has given to modulate both the transparency windows simultaneously. For some applications, it may be desirable to select only the region of a particular transparency window and control its dispersion properties. In this context, an independent control of transparency window could be very significant for designing devices in the desired frequency range. However, practically it has been a very difficult task to tune each transparency window individually. Only few theoretical efforts using hybrid graphene metal structures have been reported in this context^49,50^. But the practical realization of these structures is a difficult task due to the challenges in fabrication and electrical tuning of graphene based resonators. Further, low absorption features in graphene structures makes it difficult to observe these effects in experiments. On the other hand, metallic metamaterial structures not only exhibit strong absorption features, but also can be easily fabricated using convention photolithography techniques. In our work, we have addressed the issues related to the independent modulation of transparency window. The novelty of our paper stems from the fact that an independent modulation of transparency windows in a multi-band transparency regime is experimentally investigated and results have been separately confirmed through theory and simulations.

In this article, we experimentally and numerically examine a MM structure comprising a CW surrounded by a pair of asymmetric double C resonators (DCRs) that exhibits multi-band EIT effect in the terahertz frequency regime. We have chosen this simple but effective metal MM design to overcome the fabrication complexities as well as to experimentally demonstrate the concept of independent modulation of transparency windows. The MM structure exhibits multi-band EIT effect due to the coupling between bright CW and pair of asymmetric dark resonators i.e. left DCR and right DCR. An independent modulation of the desired transparency window is achieved by displacing one DCR with respect to the CW while keeping the other one DCR fixed at its positon. Further, a strong linear dispersion within the two transparency windows results in higher group refractive index and slow group velocity in the proposed MM structure. Further, we have employed coupled harmonic oscillator based theoretical model to demonstrate the comprehensive understanding of independent modulation aspect in the context of our study. The model also helps us to retrieve information on coupling coefficients with respect to modulating transparency windows. The paper is organized as follows: In the first section, we provided the design, fabrication and experimental details of the MM structure. We have also discussed the evolution of multi-band EIT windows in the proposed structure in this section. In the next section, we discuss the independent modulation behavior of multi-band EIT windows through simulation as well as experiments. The dispersion property and slow light effect in our proposed MM configuration have also been discussed in this section. The analytical model for the multi-band EIT effect is discussed in the next section followed by the summary in the last section.

## Design and Experimental Details

**Figure 1:**
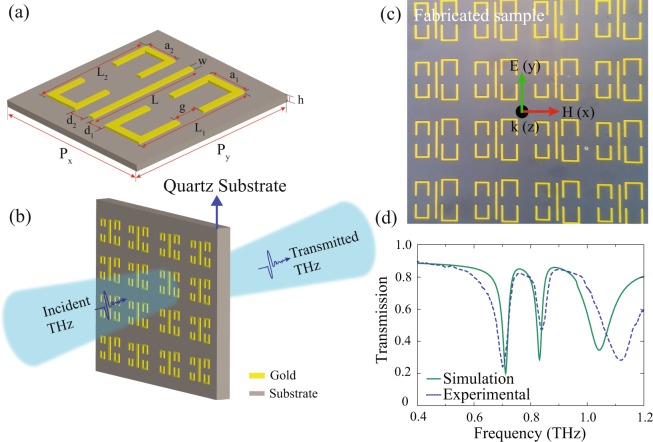
(**a**) Unit cell of the proposed metamaterial structure comprising of a CW and two asymmetric DCRs. The geometrical parameters are: *P*_*x*_ = *P*_*y*_ = 144, *h* = 500, *L* = 88, *w* = 4, *L*_1_ = 100, *L*_2_ = 88, *a*_1_ = 40, *a*_2_ = 34, *d*_1_ = *d*_2_ = 10 *μm*. (**b**) Schematic depicting terahertz transmission through the proposed metamaterials geometry. (**c**) shows optical microscope image of the fabricated sample, and (**d**) depicts plot of the experimentally and numerically calculated terahertz transmission spectra of the proposed MM structure for the y-polarized incident light. Solid lines represent the simulated, while dotted line corresponds to the measured transmission spectra.

The schematic illustration for the investigation of the multi-band EIT effect in the proposed planar metamaterial structure is shown in Fig. 1. The unit cell of the proposed MM structure comprises of a pair of asymmetric DCRs and the CW, made up of gold having a thickness of *t* = 200 *nm*, placed on a milky quartz substrate having dielectric permittivity of 4.68, periodicity, *P*_*x*_ = *P*_*y*_ = 144 *μm* and thickness, *h* = 500 *μm*, is shown Fig. 1(a). The CW has length, *L* = 88 *μm* and width, *w* = 4 *μm*. The asymmetric DCRs, placed symmetrically on both side of the CW have same width, *w* = 4 *μm*. The left DCR has length, *L*_2_ = 88 *μm* and width, *a*_2_ = 34 *μm* while the right DCR has length, *L*_1_ = 100 *μm* and width, *a*_1_ = 40 *μm*. The vertical gap between the pair of C resonators in both the DCRs are kept fixed at *g* = 20 *μm*. The distance between the CW and right DCR is termed as *d*_1_ while the distance between the CW and left DCR is termed as *d*_2_. Figure 1(b) represents the experimental schematic of the proposed MM structure. The optical micrograph image of the fabricated sample is shown in Fig. 1(c). For the fabrication of the samples, electron beam lithography has been used in the clean room environment. The samples were fabricated on a milky quartz substrate having permittivity of 4.68 and thickness of 500 *μm*. For measurement commercially available terahertz Toptica system (Terahertz frequency domain spectroscopy) has been used. This system has high frequency resolution of 2 MHz. In this system terahertz radiation are being generated and detected in frequency domain using photo-conducive antenna. In order to generate terahertz radiation, the photo mixer antennas are being illuminated by two diode lasers having frequencies approximately close to each other. By measuring photo current at the photo-conductive antenna based detector, terahertz radiation can be detected. To resolve the spectral response of the MM sample, the frequency was swept from 50 GHz to 1220 GHz in 40 MHz step size and integration time was three miliseconds. To reduce absorption of terahertz radiation by water vapour, the measurement was carried at room temperature and dry nitrogen atmosphere. In order to initially have an idea about the design of metamaterials samples and further elucidate the experimental results, we used commercially available simulation software called CST Microwave Studio. The numerically simulated and the experimentally measured transmission spectra of the MM structure are represented by the solid and the dotted lines, respectively, in Fig. 1(d). From the figure, it is evident that the experimentally measured transmission spectra through the samples matches well with the numerically simulated one.

**Figure 2:**
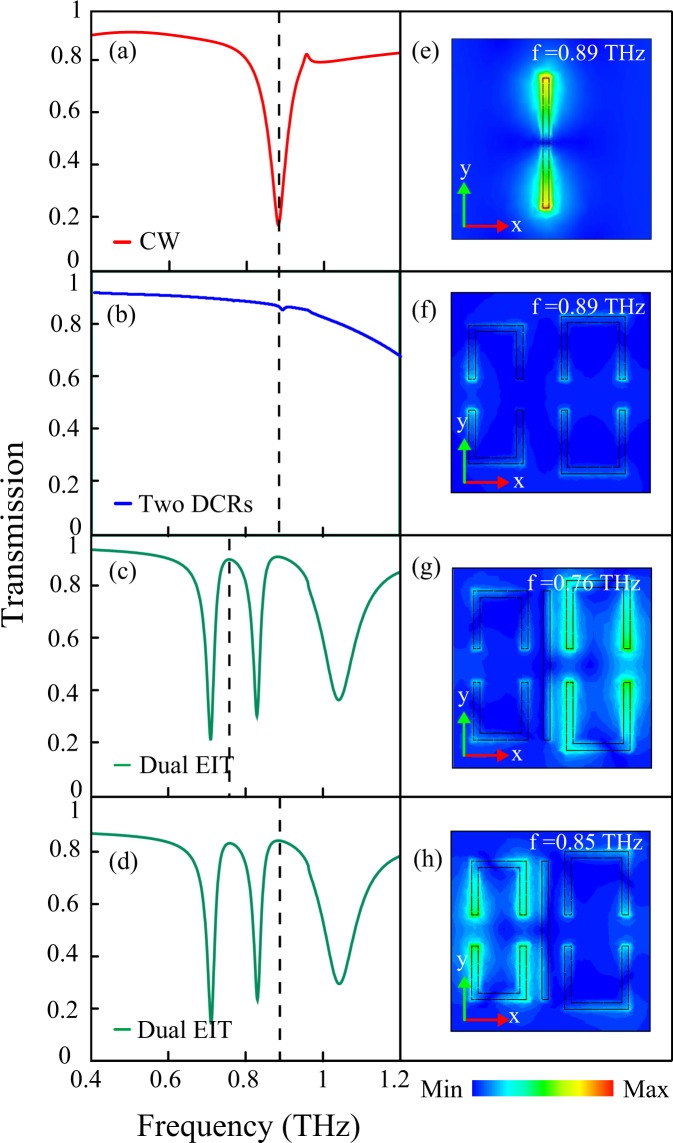
Terahertz transmission through the CW (**a**), the two asymmetric DCRs (**b**), the proposed MM configuration (**c**,**d**). (**e**–**h**) Show electric field profiles for the cut wire (at 0.89 *THz*), two asymmetric DCRs (at 0.89 *THz*), transmission peak 1 (at 0.76 *THz*) and transmission peak 2 (at 0.88 *THz*), respectively. Polarization direction of incident electric field is represented by green arrow.

In order to understand the evolution of the multi-band EIT effect in the MM structure, we have examined the terahertz transmission response as well as induced electric field profiles in the CW, two DCRs and combined MM structure (see Fig. 2). The incident terahertz light is polarized along the y-direction which is represented by the green arrow in the figure. The red traces in Fig. 2(a) signify the transmission response of the CW. It can be seen that the CW is excited directly by the incident electric field and exhibits a dipolar resonance at 0.89 *THz*. This is also apparent from the induced electric field profile of the CW at the resonance frequency shown in Fig. 2(e). Thus, the CW behaves as a bright mode. The transmission spectrum corresponding to the pair of DCRs is shown in Fig. 2(b) (blue traces) and corresponding field profile is shown in Fig. 2(f)). In contrast to the CW, the left and right DCRs are not directly excited by the incident electric field. This is because of the orientation of the two DCRs and their gaps which are perpendicular w.r.t. the incident electric field. Therefore, DCRs act as the dark modes. In the case of the combined structure shown in Fig. 2(c), the near field coupling between the CW and DCRs excites the pair of dark DCRs on both sides of the CW. Then, the destructive interference between the bright dipolar and dark DCRs excitations leads to the multi-band EIT effect in the proposed metamaterial structure. Because of asymmetry in the sizes of DCR pairs, we get two distinct transparency peaks at frequencies at 0.76 *THz* and 0.89 *THz* marked by dotted lines in Fig. 2(c,d), respectively. The electric field profiles at the first and the second peak of the two transparency windows are shown in Fig. 2(g,h), respectively. It may be observed in Fig. 2(g) that the electric field is confined at the right DCR at 0.76 *THz*, whereas, it is confined in the left DCR at 0.89 *THz*. The excitation of multi transparency window is also accompanied by the suppression of dipolar excitation.

## Independent Modulation of Multi-Band Transparency Windows

**Figure 3:**
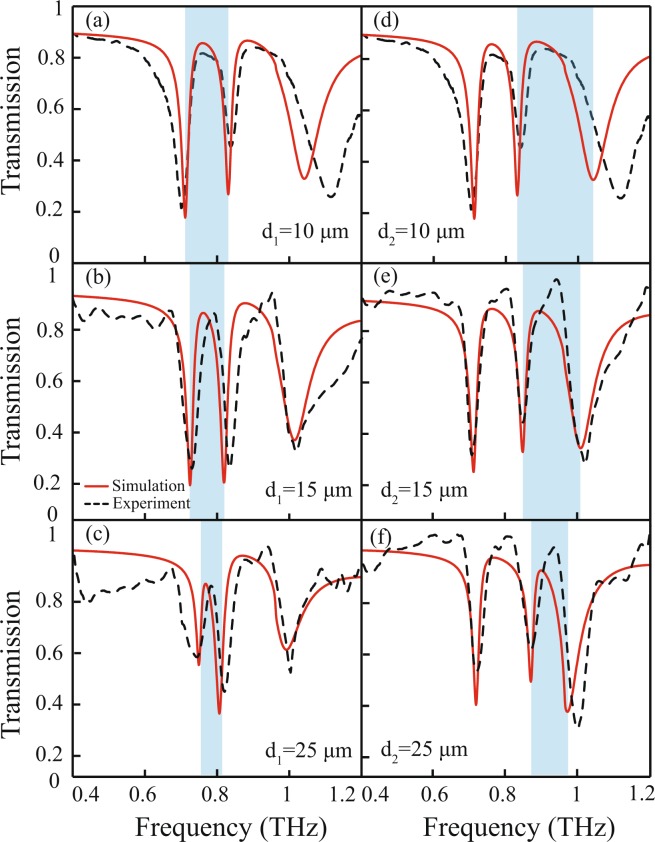
Simulated and experimentally measured terahertz transmission for various separation values ‘*d*_1_’ and ‘*d*_2_’ between the CW and the pair of DCRs. (**a**–**c**) represents transmission for *d*_1_ = 10  *μm*, 15 *μm* and 25 *μm* respectively, whereas (**d**–**f**) correspond to *d*_2_ = 10 *μm*, 15 *μm* and 25 *μm* respectively. The modulation in the transparency windows is reflected by the blue shaded color.

In our work, we have investigated independent modulation of transparency window in a system capable of exhibiting multiple transparency windows through experiments and supported results with numerical simulations and theory. The independent modulation of the EIT transparency windows will provide us the flexibility to choose the desired frequency for device designing and construction. In Fig. 3, we show experimentally and numerically, the independent modulation of the transparency windows by varying the near field coupling between the CW and DCRs. The red traces represent simulated transmission spectra and corresponding experimentally measured spectra is indicated by the dashed traces. For the modulation of the 1^*st*^ window, distance, *d*_1_ is varied from 10 *μm* to 25 *μm* to vary near field coupling between CW and right DCR. For *d*_1_ = 10 *μm*, the 1^*st*^ EIT window varies from 0.71 *THz* to 0.83 *THz*. As *d*_1_ increases to *d*_1_ = 25 *μm*, the window narrows down to 0.75  *THz* to 0.80 *THz*. In this modulation process, the left DCR is kept fixed at its position. Similarly, the independent modulation of 2^*nd*^ transparency window is investigated by changing the distance of the left DCR w.r.t. the CW (as shown in Fig. 3(b)). In the figure, the red traces for *d*_2_ = 10 *μm* corresponds to the most widest 2^*nd*^ EIT window ranging from 0.83 *THz* to 1.0 *THz* (shown by blue shaded area). The window narrows down to 0.87 *THz* to 0.97 *THz* for *d*_2_ = 25 *μm*. It is worth mentioning that the peak frequencies of the transparency windows remain almost constant during the modulation process. Also our experimentally measured transmission spectra are in good agreement with the simulations.

**Figure 4:**
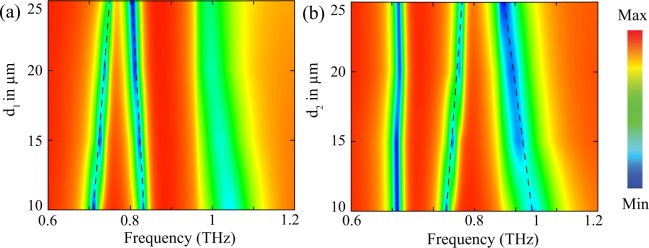
Contour plot of numerically simulated  *THz* transmission for different distance between CW and DCRs. Color bar shows the magnitude of transmission intensity. (**a**) Represents contour plot for the variation of 1^*st*^ transparency window with distance between CW and right DCR (**b**) represents contour plot for the variation of 2^*nd*^ transparency window with distance between CW and left DCR respectively.

In order to present a comprehensive picture of the independent modulation of the multi-band EIT effect, we have also plotted the contour and color plots for various displacements of right (*d*_1_) and left DCRs (*d*_2_) w.r.t. CW. The contour plots are shown in Fig. 4(a,b), respectively. In the figures, the frequency and displacement are drawn along x and y-axis and the strength of the signal is indicated through the color bar. The modulation of 1^*st*^ transparency window is apparent in Fig. 4(a) while the 2^*nd*^ transparency window remains almost unchanged. Again in Fig. 4(b), the modulation of 2^*nd*^ EIT window is quite evident. Therefore, one can modulate the desired transparency window by keeping one DCR resonator fixed and displacing the other w.r.t. the CW in our proposed configuration. The feature could be useful in achieving selectively tunable response of the multi-band EIT effect.

In EIT, we get transparency region in an otherwise absorptive region which is accompanied by the steep dispersion within the transparency window. A strongly dispersive media can cause the group velocity of light to slow down which has applications in enhanced nonlinear effects, pulse delay, optical buffers, etc.^51–56^. In order to demonstrate practical applicability of our study we further examine how these structures can result in strong dispersion within the transparency windows and can cause the light to slow. For doing so, we have calculated group velocity in two transparency windows and plotted them with terahertz frequency. Although there have been reports mentioning the application of electromagnetically induced transparency for slow light systems, however here we explicitly perform these calculations and show slow light behavior which can also be independently tuned.

**Figure 5:**
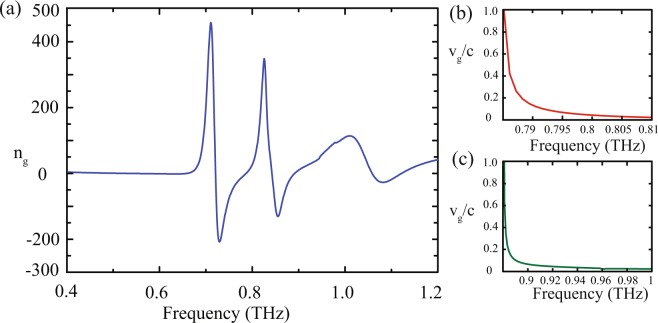
(**a**) Numerically calculated group refractive index versus frequency (**b**) and (**c**) depicts variation of group velocity versus frequency in the normally dispersive regimes of the 1^*st*^ and 2^*nd*^ transparency windows, respectively.

The stronger phase dispersion occurs in the vicinity of the transparency windows and results in a larger group index (*n*_*g*_) which is expressed as

1 $${n}_{g}=\frac{c}{h}\frac{d\phi }{d\omega }$$

where, c is the velocity of light in free space, h is the substrate thickness and *ϕ* is the transmission phase of the MM structure. In Fig. 5(a), we have plotted group index value as a function of frequency to have a quantitative appreciation of the group index values within the transparency windows. It is evident from the figure that the normal dispersion of the group index is accompanied by anomalous dispersion around the transparency windows. In order to understand the slow light property of the light passing through the proposed MM structure, we plot normalized group velocity (*v*_*g*_/*c*) versus frequency in normally dispersive regions. The results are shown in Fig. 5(b,c). Figure 5(b) depicts the variation of the group velocity versus frequency in the range of 0.78 *THz* to 0.81 *THz* falling within the 1^*st*^ transparency window. One may notice that group velocity decreases with an increase in frequency and ultimately becomes zero at 0.81 *THz*. Similarly, we see a reduction in the group velocity within the frequency range of 0.89 *THz* to 1.0 *THz* of the 2^*nd*^ transparency window as shown by the green traces in Fig. 5(c). We observe more steep group velocity reduction in 2^*nd*^ window compared to the 1^*st*^ window. The results clearly indicate a slow light phenomenon at the multi-band regimes of the proposed MM configuration. One can also control the slow light behaviors at a specific frequency based upon the structural parameters of the MM configuration.

## Theoretical Modelling Based on Coupled Harmonic Oscillator Systems

In order to further understand and validate numerical and experimental findings on multi-band transparency effect and independent modulation of transparency window, we employ a theoretical model based on coupled harmonic oscillator systems. One can write equations of motion for the system as^57^

2 $$\begin{array}{rcl}{x^{\prime\prime} }_{1}(t)+{\gamma }_{1}{x^{\prime} }_{1}(t)+{\omega }_{1}^{2}{x}_{1}(t)-{\Omega }_{2}^{2}{x}_{3}(t) & = & \frac{{F}_{1}}{{m}_{1}}{e}^{-i\omega t}+c.c\\ {x^{\prime\prime} }_{2}(t)+{\gamma }_{2}{x^{\prime} }_{2}(t)+{\omega }_{2}^{2}{x}_{2}(t)-{\Omega }_{1}^{2}{x}_{3}(t) & = & 0\\ {x^{\prime\prime} }_{3}(t)+{\gamma }_{3}{x^{\prime} }_{3}(t)+{\omega }_{3}^{2}{x}_{3}(t)-{\Omega }_{2}^{2}{x}_{1}(t)-{\Omega }_{1}^{2}{x}_{2}(t) & = & 0\end{array}$$

where *γ*_1_, *γ*_2_, *γ*_3_ and *ω*_1_, *ω*_2_, *ω*_3_ are the damping factors and resonance frequencies of the CW, right DCR and left DCR and *ω* is the frequency of the incident terahertz light. Ω_1_ and Ω_2_ represents coupling coefficient between CW and two DCRs. Assuming the solution of Eq. (2) as

3 $${x}_{j}={N}_{j}{e}^{-i\omega t}+c.c$$

the energy dissipated by the MM structure exhibiting multi-band EIT effect is given as

4 $$P(\omega )=\frac{{D}_{2}{D}_{3}-{\Omega }_{r}^{4}}{{D}_{1}({D}_{2}{D}_{3}-{\Omega }_{r}^{4})-{D}_{2}{\Omega }_{c}^{4}}$$

where *D*_1_ = *ω*_1_^2^ − *ω*^2^ − *iωγ*_1_, *D*_2_ = *ω*_2_^2^ − *ω*^2^ − *iωγ*_2_, *D*_3_ = *ω*_3_^2^ − *ω*^2^ − *iωγ*_3_.

The transmission amplitude for the system is given by the expression

5 $$t(\omega )=1-{\text{Im}}P(\omega )$$

**Figure 6:**
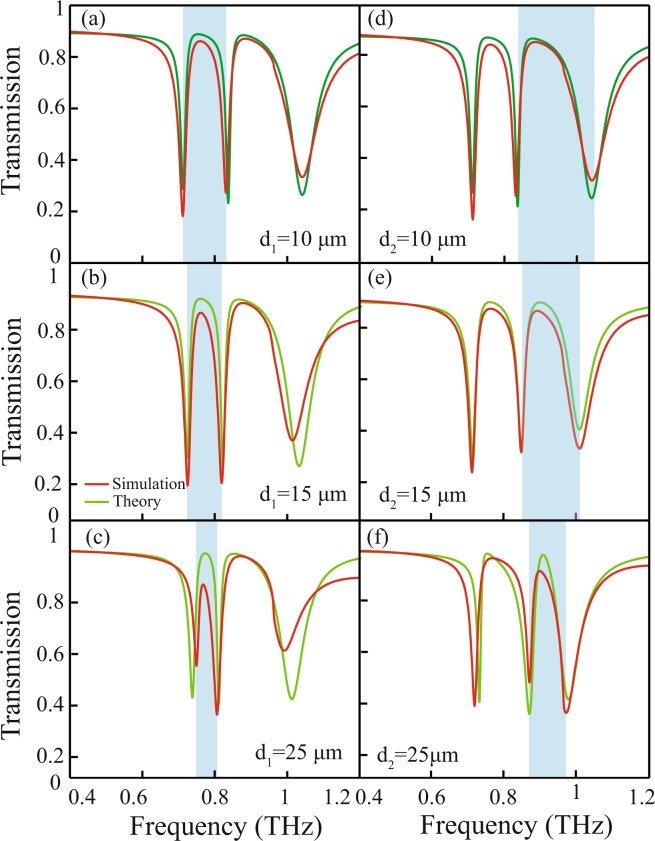
Simulated and theoretically fitted transmission spectra (**a**–**c**) for various distance (*d*_1_) between the CW and right DCR for the modulation of 1^*st*^ transparency window and (**d**–**f**) for various distance (*d*_2_) between CW and left DCR for the modulation of the 2^*nd*^ transparency window of the proposed multi-band EIT metamaterial.

One can use Eq. (5) to theoretical fit the numerically obtained transmission response. In Fig. 6, we have shown theoretically fitted transmission spectrum which indicates reasonably good agreement with the numerical simulations. The solid red traces represent numerical simulations for various *d*_1_ and *d*_2_, while the solid green traces represent the corresponding theoretically fitted transmission spectrum.

**Figure 7:**
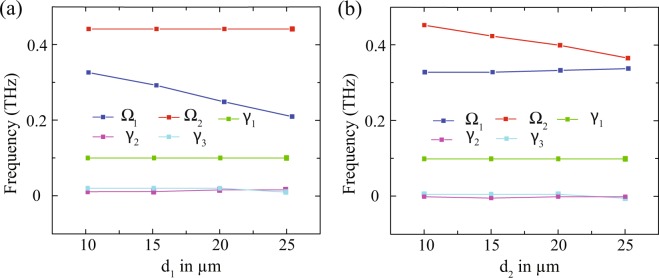
The variations of the fitting parameters *γ*_1_, *γ*_2_, *γ*_3_, Ω_1_ and Ω_2_ as the function of the (**a**) distance between CW and right DCR (*d*_1_) for the modulation of 1^*st*^ transparency window (**b**) distance between CW and left DCR (*d*_2_) for the modulation of the 2^*nd*^ transparency window of the multi-band EIT spectrum.

In order to understand the mechanism involved in modulation, we plot fitting parameters *γ*_1_, *γ*_2_, *γ*_3_, Ω_1_ and Ω_2_ versus displacements ‘*d*_1_’ and ‘*d*_2_’ responsible for the coupling strength. The results are shown in Fig. 7. The variations of the fitting parameters versus displacement *d*_1_ are shown in Fig. 7(a). One may note that the coupling term Ω_2_ remains almost constant. This is due to the fact that coupling strength between the CW and left DCR do not change since *d*_2_ remains constant in this case. On the other hand, coupling term Ω_1_ decreases from 0.32 *THz* to 0.2 *THz* because of decrease in coupling strength with an increase in *d*_1_. The damping parameters *γ*_1_, *γ*_2_, *γ*_3_ do not vary with displacement as they are solely responsible for the loss associated with the modes. Similarly, we study the variation of the fitting parameters with displacement *d*_2_ and results are shown in Fig. 7(b). In this case, coupling term Ω_1_ remains almost constant, however Ω_2_ decreases from 0.45 *THz* to 0.36 *THz*. In this case, *d*_1_ remains unchanged resulting in same coupling strength, however a change in Ω_2_ reflects a change in coupling strength because of variation in *d*_2_. Again the fitting parameters for damping coefficients remains unchanged in this case also. Therefore, one can control the coupling strength by varying the displacement between CW and resonators to achieve a desirable modulation response. Even though the experimental measurements have been independently manifested by numerical simulations, we employed coupled harmonic oscillator based theoretical model to comprehensively understand the independent modulation aspect which has been missing in the works reported so far. As evident from the results, coupling coefficients are found to vary corresponding to their respective modulating window which has been quantitatively discussed.

## Conclusions

A coupled terahertz metamaterial comprising of a CW and a pair of asymmetric DCRs exhibiting a multi-band EIT effect is numerically and experimentally investigated. The evolution of multi-band EIT effect in the MM structure is achieved via near field coupling between the CW and DCRs. We demonstrate a novel method of modulating the transparency windows independent of each other in the proposed metallic MM configuration. This is achieved by displacing one DCR w.r.t the CW while keeping the other fixed. As the separation of one DCR from the CW increases, the coupling between them decreases, leading to the narrowing of one transparency window, however other remains unchanged. We analyzed strongly dispersion characteristics of the transparency windows as well as the associated slow light effect. It is observed that the group velocity reduces by a factor of 0.2*c* at 0.79 *THz* in the 1^*st*^ transparency region. In order to understand the mechanism of independent modulation as well as validate our numerical and experimental findings, we employed a mechanical model based on coupled harmonic oscillator system. The model shows that the modulation of the 1^*st*^ and 2^*nd*^ transparency windows are achieved due to the variation of the coupling terms Ω_1_ and Ω_2_ respectively. The model predicts terahertz transmission spectrum in a good agreement with experiments and simulations. Our study is vital in the development of multi-band slow light devices, buffers, modulators, etc.

## Methods

The MM samples were fabricated by using conventional electron beam lithography technique in the clean room environment. The measurements were performed using commercially available terahertz Toptica system (teraherz frequency domain spectroscopy) consisting of fiber coupled photo-conductive antenna based emitter and detector. For numerical simulations, we have employed finite element frequency domain solver in CST Microwave Studio.
